# Captopril Polyvinyl Alcohol/Sodium Alginate/Gelatin-Based Oral Dispersible Films (ODFs) with Modified Release and Advanced Oral Bioavailability for the Treatment of Pediatric Hypertension

**DOI:** 10.3390/ph16091323

**Published:** 2023-09-19

**Authors:** Hamdy Abdelkader, Jelan A. Abdel-Aleem, Heba Salah Mousa, Marwa O. Elgendy, Adel Al Fatease, Heba A. Abou-Taleb

**Affiliations:** 1 Department of Pharmaceutics, College of Pharmacy, King Khalid University, Abha 62223, Saudi Arabia; afatease@kku.edu.sa; 2 Department of Industrial Pharmacy, Faculty of Pharmacy, Assiut University, Assiut 71526, Egypt; jelan.abdelrazik@pharm.aun.edu.eg; 3 Pharmaceutical Analytical Chemistry Department, Faculty of Pharmacy, South Valley University, Qena 83523, Egypt; heba.salah@svu.edu.eg; 4 Department of Clinical Pharmacy, Beni-Suef University Hospitals, Faculty of Medicine, Beni-Suef University, Beni Suef 62521, Egypt; marwaosamaelgendy@yahoo.com; 5 Department of Clinical Pharmacy, Faculty of Pharmacy, Nahda University (NUB), Beni Suef 62521, Egypt; 6 Department of Pharmaceutics and Industrial Pharmacy, Faculty of Pharmacy, Merit University, Sohag 82755, Egypt; heba.ahmed@merit.edu.eg

**Keywords:** captopril, pediatric, hypertension, oral mucosa, oral films

## Abstract

Hypertension can begin in childhood; elevated blood pressure in children is known as pediatric hypertension. Contrary to adult hypertension, there is a scarcity of commercial medications suitable for the treatment of pediatric hypertension. The aim of this study was to develop orally dispersible films (ODFs) loaded with captopril for the treatment of hypertension in children. Captopril-loaded ODFs were composed of different blends of synthetic polymers, such as polyvinyl alcohol (PVA) and polyvinyl pyrrolidone, and natural polymers, such as sodium alginate (SA) and gelatin. The ODFs were characterized based on their mechanical and thermal properties, drug content, surface morphology, in vitro disintegration, in vitro release, and bioavailability. A novel HPLC method with precolumn derivatization was developed to precisely and selectively determine captopril levels in plasma. A low concentration of PVA and a high concentration of SA generated ODFs with faster hydration and disintegration rates. SA-based films exhibited fast disintegration properties (1–2 min). The optimized modified-release film (F2) showed significant (*p* < 0.05) enhancement in bioavailability (AUC = 1000 ng min/mL), with a value 1.43 times that of Capoten^®^ tablets (701 ng min/mL). While the plasma concentration peaking was in favor of the immediate-release tablet, T_max_ was significantly prolonged by 5.4 times for the optimized ODF (3.59 h) compared with that of the tablets (0.66 h). These findings indicate uniform and sustained plasma concentrations, as opposed to the pulsatile and rapid plasma peaking of captopril from the immediate-release tablets. These findings suggest that the modified release of oral films could offer more favorable plasma profiles and better control of hypertension than the conventional release tablets.

## 1. Introduction

Hypertension, or elevated blood pressure, is a significant medical condition that affects 1.28 billion adults aged 30–79 globally [1] and can increase the risk of heart, kidney, and brain disorders [2]. Hypertension can begin in childhood; elevated blood pressure in children is called pediatric hypertension [2]. The prevalence of hypertension in school children at the age of 13 has been reported to be 2% to 4% of this population by the American Academy of Pediatrics [3]. In younger children (<6 years), hypertension is the result of kidney diseases [2]. Pediatric hypertension has been correlated with coronary artery diseases in adults [4]. Therefore, the treatment of pediatric hypertension is strongly recommended by pediatricians. Many pharmacological drug classes have been approved for the treatment of hypertension, including captopril. Captopril is recommended for the long-term therapy of pediatric hypertension [2,5].

Captopril is a prototype angiotensin-converting enzyme (ACE) inhibitor that is commercially available under the name Capoten in 12.5, 25, and 50 mg tablets. It was developed at Squibb (Smith and Vane, 2003) [6]. Captopril is a freely soluble white crystalline powder; it is safe and efficient for long-term hypertension therapy [7]. It is recommended to start with the lowest dose in order to attain high therapeutic benefits with minimal or no adverse effects. Captopril undergoes extensive hepatic metabolism, and its oral bioavailability in dogs has been reported to be 30–40% [8]. Food can significantly reduce the bioavailability of captopril by 35–55% [9]. Our hypothesis is to modify the release of the antihypertensive drug captopril and reduce rapid plasma peaking after oral administration to optimize the therapy for more uniform plasma drug levels and to reduce spiking and unwanted, concentration-related effects [10]. However, the conventional extended-release matrix systems may end up with a delayed response and poor therapeutic outcomes [11].

While plenty of commercial dosage forms are available for the treatment of adult hypertension, when it comes to pediatric hypertension, the only available dosage forms are either extemporaneously prepared liquid preparations from commercial tablets or commercial liquid dosage forms (captopril syrups). These dosage forms either have very short shelf lives or are difficult to adjust to the correct dosage. Further, these conventional dosage forms do not offer optimum plasma levels of antihypertensive drugs due to rapid absorption and fluctuating plasma levels. Modified/sustained release of antihypertensive drugs can offer smoother plasma concentrations with more uniform and consistent plasma levels, good peak-to-trough ratios, and reduced side effects [10]. Poor patient adherence to medication is a well-acknowledged problem; among children, this problem is even more common and widespread [12]. Therefore, modified/sustained release systems can prolong drug release and reduce the need for frequent dose administration [10].

Alternatives to the standard systemic oral solid dose forms, such as tablets, coated tablets, and capsules, include oral mucosal drug delivery devices. These alternative oral mucosal delivery systems, such as oral films, offer disintegration and dissolution in the buccal cavity, and the absorption of drugs initiated from buccal mucosa can allow the drugs to reach the blood vessels while circumventing first-pass metabolism [13]. This route of drug delivery has been successful for many cardiovascular active pharmaceutical ingredients, such as nitroglycerin, verapamil, and propafenone [14,15]. Oral films can be suitable in emergencies where water to aid in ingesting the drug is not necessarily required and in where limited access to water is occasionally experienced, such as in emergencies or while traveling [16].

Oral dissolving/dispersible films (ODFs) can offer a potential advantage for the special patient population that can experience difficulty in swallowing solid dosage forms, such as pediatric and elderly patients [16]. Even in adult populations, the need to swallow a tablet has been reported to be a reason for 8% of patients to show poor adherence to the medicine and 4% to stop taking the medicine completely [16]. For example, atenolol-based oral films composed of hydroxypropyl methylcellulose, sodium alginate, and carboxymethyl cellulose sodium were prepared as a more convenient alternative to tablets for elderly patients with hypertension [17]. More recently, ODFs have attracted growing attention as a potential dosage form for pediatrics because they can solve various administration-compliance-related issues, such as choking risk, palatability, and instability problems inherent to liquid dosage forms [18]. Among the excipients successfully used to prepare ODFs for pediatric uses and ensure rapid disintegration (up to 180 s) are gelatin, hydroxypropyl methylcellulose, pullulan, and pectin [18,19].

Gelatin is a commonly used biomaterial in pharmaceutical and confectionary industries for desirable, safe, and controlled-release forming properties [20]. It has been successfully used alone (plasticized with glycerin) or combined with other cellulose polymers to form oral films [21,22]. Gelatin has been investigated for sustained release properties; it can sustain drug release by entrapping drug molecules in the polymer’s crosslink gap [23].

In this study, various polymers such as polyvinyl alcohol, sodium alginate, and gelatin were employed; these selected polymers are commonly used for their film-forming properties, super-disintegration, and availability in the food and pharmaceutical industries, respectively [24,25,26]. Sucralose was used as a sweetening agent with a super sweetening power of over 600 times the sucrose of table sugar [27]. Gelatin and the film-forming polymer polyvinyl alcohol were combined to study how these two substances affect oral bioavailability, drug release, and disintegration time in vitro. Furthermore, a novel precolumn derivatization technique was adopted to impart chromophores to captopril, and a new HPLC method was developed, enabling its analysis in plasma.

## 2. Results and Discussion

One of the most popular methods for casting oral films is the solvent casting technique because of its simplicity, scalability, and suitability for various classes of drugs due to its mild preparation conditions and non-involvement of heat [16]. The solvent casting technique was used to successfully create four distinct captopril ODFs. These four distinct formulations were made up of various mixtures of the film-forming polymers: gelatin, sodium alginate, and polyvinyl alcohol (PVA). Two different concentrations of PVA (10% and 20%) and three different concentrations (20%, 32%, and 53%) of gelatin were used, as shown in Table 1. Glycerin was used with the ODFs. It is one of the most efficient plasticizers for its small molecular size, water-binding capacity, and miscibility with various polymeric strands [28]. Its concentration was fixed at 23% in all the prepared film formulations. A concentration range of 20 to 30% has been reported as sufficient for plasticizing the polymeric films [28,29]. A greater percentage of glycerin has been reported to produce gelatin-based films with poor quality [30].

Table 1 lists the prepared captopril ODFs’ dimension, in vitro disintegration time, surface pH, drug content, and folding endurance. The thickness of the prepared films ranged from 380 to 500 µm and the weight was 38.5 to 50 mg. Although all formulations had the same amount of drug and solid weight of gelatin and different polymers (PVA and SA), the generated ODFs showed marked, significant differences (*p* < 0.05) in thickness and weight. This can be ascribed to the spreading capacity and contractility of the resulting polymeric matrix formed during the evaporation and casting of the polymeric film. For example, F2 and F3 showed relatively larger thickness and greater weight than F1 and F5.

The drug contents of the prepared captopril ODFs were in the range of 11–12.5 mg. This range is worth almost half of the commercially available immediate-release tablet products available for the treatment of adult hypertensive patients on the Egyptian pharmaceutical market. This could indicate that the optimized ODF is suitable for children’s dosage regimen. The surface pH was 7.0 to 7.5, which is physiologically compatible. Folding endurance recorded for the prepared captopril ODFs ranged from 2 to 10. The higher the number recorded, the better the mechanical properties that can be attributed to the ODF. Therefore, poor mechanical properties were attributed to F4 (the highest gelatin concentration) [30].

The in vitro disintegration time was between one and ten minutes. F1 and F2 demonstrated superior in vitro disintegration compared with the other prepared captopril ODFs (F3 and F4). A low concentration of PVA and high concentration of SA generated ODFs with faster hydration and disintegration rates. Sodium alginate (SA) has super-disintegrating properties and generates films with fast disintegration properties [25]. Faster disintegration is a regulatory requirement for ODFs [31,32]. Longer disintegration time was attributed to F3 and F4, which contained a higher concentration of PVA (20%) and 0% of SA, respectively. PVA has good film-forming characteristics, although its larger concentrations negatively extend the time until disintegration [24]. This could be ascribed to poor hydration and dissolution of PVA. Therefore, F2 with an acceptable disintegration time (≤ 1 min) was selected for pharmacokinetic studies [33].

### 2.1. FTIR and DSC Studies

**Figure 1:**
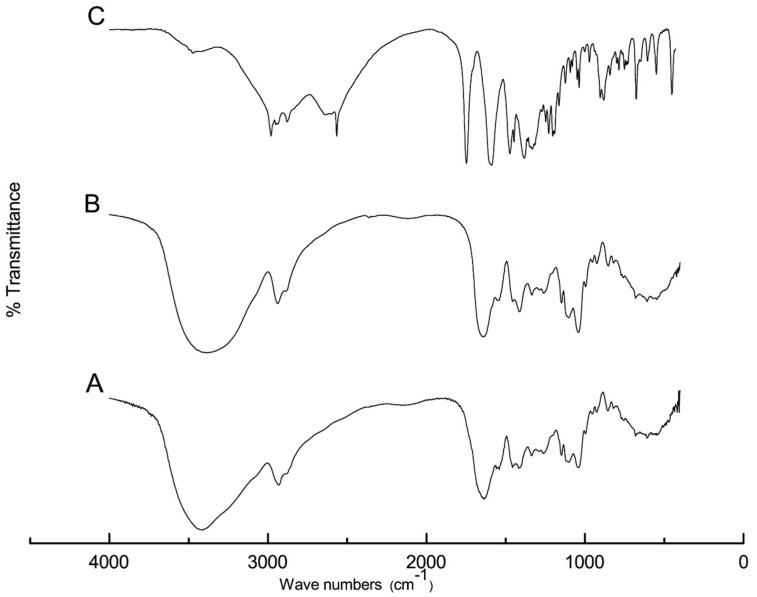
FTIR spectra of captopril (A), drug-free F2 (B), and captopril-loaded F2 (C).

Figure 1 shows the FTIR spectra of captopril powder, F2, and drug-free F2. Two characteristic peaks of captopril at 2980 and 2565 cm^−1^ were attributed to -OH (stretching) and -SH (stretching), respectively [34]. Another two strong double peaks appeared at 1750 and 1590 cm^−1^ and were assigned to the carbonyl group (-C=O) [34], as shown in Figure 1A.

Figure 1B displays the FTIR spectrum of the drug-free F2 that contained sucralose; glycerol; and a mixture of PVA, gelatin, and SA. Broad peak appeared at 3600–3400 assigned for H-C and H-O- stretching; the band assigned to the C-H asymmetric stretching vibration, was at 2927 cm^−1^ [26]. Figure 1C shows the spectrum of captopril-loaded F2, which demonstrates the complete disappearance of the characteristic captopril peaks, indicating possible H-bond formation and interactions with –COOH and -OH of sodium alginate and PVA, respectively.

**Figure 2:**
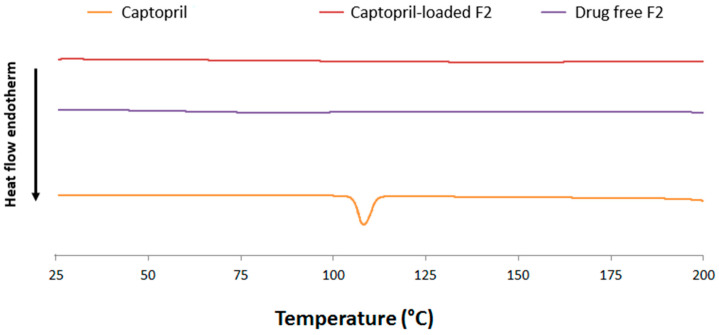
DSC thermogram of captopril alone, drug-loaded F2, and drug-free F2.

DSC thermograms of captopril, drug-free F2, and captopril-loaded F2 are shown in Figure 2. A characteristic melting peak of captopril appeared at 110 °C [6]. Drug-free films did not show any thermal events as they are composed of amorphous polymers. More interestingly, the captopril-melting peak disappeared from F2, indicating that the drug was dispersed in an amorphous/soluble form within the polymeric matrix. These results are in accordance with those obtained from FTIR studies.

### 2.2. In Vitro Captopril Release

**Figure 3:**
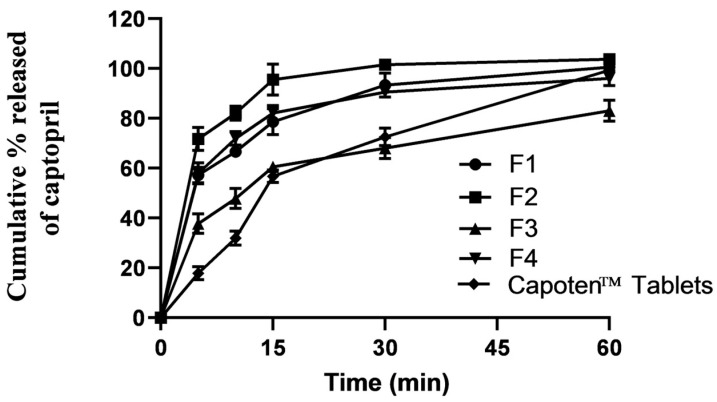
In vitro release profiles of captopril from the prepared oral films and commercial tablets (Capoten).

In vitro captopril release profiles obtained from the prepared four captopril ODFs were compared with those obtained from the commercially available captopril tablets (Capoten^®^), as shown in Figure 3. The in vitro release study was conducted in a simulating saliva fluid containing 0.1% Tween 80, pH was adjusted at 7, and the temperature was 37 °C.

Generally, all the prepared captopril ODFs showed superior dissolution rates compared with the commercial tablets. This result can be ascribed to the faster disintegration time. Similar results were reported for vardenafil ODFs compared with the commercial tablets [24]. There were no marked differences among the release profiles of F1, F2, and F4. However, F3 recorded markedly slower release profiles. This could be ascribed to the longer disintegration time compared with other ODFs. Table 2 shows relative release rates (RRR) at three different points (5, 10, and 15 min) for the prepared four captopril ODFs compared with the commercial captopril tablets. The fastest release rates (>4-fold at 5 and 10 min) were recorded for F2, whilst F3 showed the lowest release rates. The general release mechanism for the prepared captopril ODFs was the Higuchi diffusion model, as evident from the best fitting linear model (R^2^) (Table 2).

### 2.3. SEM

**Figure 4:**
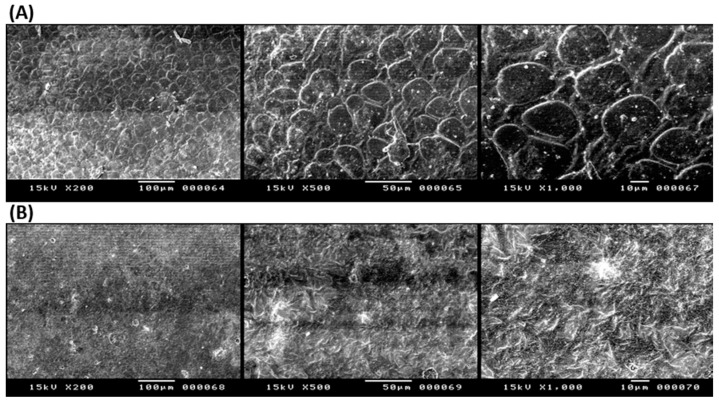
SEM micrographs at three different magnifications for (**A**) captopril ODF (F2) and (**B**) drug-free ODF (F2).

The surface morphology of some selected ODFs (F2 and drug-free F2) was studied using SEM, as shown in Figure 4A,B. Captopril F2 was studied for the morphology and homogeneity of the polymeric film formed using SEM. F2 showed smooth surfaces with honeycomb-like surfaces, as shown in Figure 4A. These honeycomb-like structures could increase the surface area of the ODF; hence, this could enhance surface drug dissolution. Drug-free F2 samples showed uniform and homogenous films at low magnifications. In contrast, at higher magnifications, needle-shaped crystals could be visible on the surface, which could be due to crystals of the sucralose (Figure 4B).

### 2.4. Pharmacokinetics Study

#### 2.4.1. Validation of the Proposed HPLC Method

Determination of captopril in plasma is a very challenging issue due to its structural features, in that it lacks any chromophores; the presence of chiral centers results in interconverting cis–trans isomers; and instability due to the susceptibility of its sulfhydryl group oxidation. This work presents a novel derivatization of captopril with FMOC-Cl, followed by its rapid HPLC determination in plasma samples. The developed HPLC method was validated according to the ICH guideline Q2 (R1) [35] and the US-FDA guidance for bioanalytical method validation [36].

##### Linearity, Limit of Detection (LOD), and Limit of Quantification (LOQ)

Calibration curves for captopril were obtained by plotting its peak area ratio and the internal standard against its corresponding concentration. Linearity data are presented in Table 3. The proposed method demonstrated good linearity with the determination coefficient (R^2^) of 0.9998, indicating good fitting of the calibration data to the regression line. The LOD and LOQ were calculated based on the signal-to-noise ratios of 3 and 10, and found to be 4.0 and 13.4 ng/mL, respectively. This indicates that the developed HPLC method has comparable sensitivity to the reported LC–MS methods without the need for an expensive LC–MS instrument. The proposed HPLC method showed adequate performance for its intended use and was successfully applied for the quantification of captopril in human plasma samples and its pharmacokinetic study. It was used to study the pharmacokinetics of captopril following a single oral dosage of 10 mg tablets in human plasma samples.

##### Intra-Day and Inter-Day, Accuracy, Precision and Extraction Recovery (ER)

The accuracy and precision of the developed HPLC method were evaluated by measuring the intra-day and inter-day (n = 6) relative standard deviations (RSDs) using three different quality control plasma samples. The %RSD values did not exceed 2.122%, as shown in Table 4, which is in accordance with the guidelines [33,34], indicating good repeatability and reproducibility of the proposed method.

The extraction recoveries of captopril were assessed for three different QC plasma samples and were found to be in the range of 98.48–102.01%, indicating good repeatability and acceptable recovery. Therefore, it can be concluded that the proposed HPLC method is reliable and feasible for the determination of captopril in plasma samples.

##### Selectivity and Robustness

The developed HPLC method was efficient in separating captopril and the IS derivatives from endogenous plasma components with no interference. No matrix effects were observed, which imparted good selectivity to the method. Furthermore, small changes in the chromatographic conditions did not affect the method’s performance, demonstrating its robustness and reliability during normal usage. 

##### Pharmacokinetics

**Figure 5:**
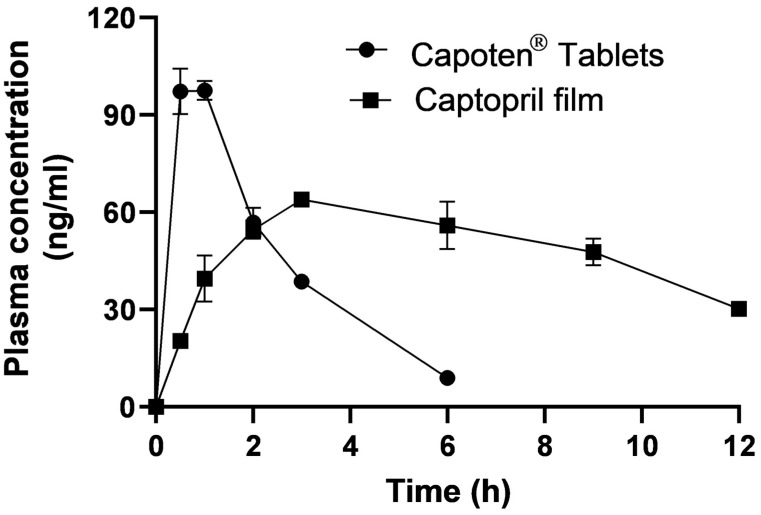
Plasma concentrations (ng/mL)–time (h) curve from captopril oral modified-release film and the commercially available immediate-release tablet (Capoten^®^). Data are presented as mean ± SD, n = 5.

Figure 5 shows plasma concentrations (ng/mL) of captopril versus time (h) from captopril oral modified-release films and commercially available immediate-release (Capoten^®^) tablets. Rapid plasma peaking (C_max_ > 100 ng/mL) followed by a sharp decline in plasma concentration was recorded for the immediate tablet form. This indicates that pulsatile therapy with a super therapeutic concentration could illicit side effects; hence, conventional tablets cannot be the ideal therapy for a chronic disease like hypertension [10]. On the contrary, slower (C_max_ = 63.66 ng/mL) but uniform and steady plasma concentration for over 4 h was obtained from the oral gelatin-based films. The optimized modified-release film (F2) showed a significant (*p* < 0.05) enhancement in bioavailability (AUC = 1000 ng min/mL) of 1.43 compared with Capoten^®^ tablets (701 ng min/mL). In contrast, the plasma concentration peaking was in favor of the immediate-release tablet; T_max_ was significantly prolonged by 5.4 times for the optimized ODF (3.59 h) compared with that of the tablets (0.66 h). These findings indicated uniform and sustained plasma concentrations compared with the pulsatile and rapid plasma peaking of captopril from the immediate-release tablets. The modified-release oral film reduced the C_max_ from 100 ng/mL to 64 ng/mL, increased the T_max_ from less than 1 h to more than 3.5 h, and increased the oral bioavailability by 1.42 (Table 5). The capacity of the prepared film to slow the absorption rate (0.66 h^−1^) and prolong plasma concentration was likely to demonstrate better control of the blood pressure [37]. The clinical practice indicated that the antihypertensive diuretic indapamide (1.5 mg) sustained-release tablets were therapeutically equivalent to the 2.5 mg immediate-release of the same drug, albeit the former showed a reduced peak plasma concentration [38]. It is worth mentioning that although the plasma data (sustained plasma levels) for F2 were not in line with the in vitro release (faster release than the commercial tablet), the only explanation could be due to possible interactions between the drug and polymeric materials, as suggested from the FTIR study.

## 3. Materials and Methods

Captopril was supplied by EIPICO Pharmaceutical Industries Co., Cairo, Egypt. Capoten^®^ tablets (batch no. 74427C) were purchased from GlaxoSmithKline (GSK) Beecham, Cairo, Egypt. Gelatin powder Type A was purchased from Alpha Chemika, Mumbai, India. Sodium alginate was purchased from Loba Chemie LTD., Mumbai, India. Glycerin, Tween 80, propranolol (IS), and fluorenylmethoxycarbonyl chloride (FMOC-Cl) were from Sigma-Aldrich (St. Louis, MO, USA). Moreover, polyvinyl alcohol was supplied by BDH Chemicals, Poole, England. Sucralose was provided by Asin Co., Mirkow, Poland.

### 3.1. Captopril Oral Dissolved Film (ODF)

Captopril ODFs were generated using the solvent casting technique. Four distinct mixes of sodium alginate (SA) (5% *w*/*v*), polyvinyl alcohol (PVA) (5% *w*/*v*), and gelatin (5% *w*/*v*) were mixed, as shown in Table 6. Glycerin and sucralose were added with fixed amounts as a plasticizer and sweetening agent, respectively. Polymeric (PVA and SA) and gelatin solutions, the plasticizer, and the sweetening agent were mixed and fully dissolved in a beaker (100 mL); then, they were transferred into a petri dish with a diameter of 9 cm. The solutions were allowed to cast out films in ambient conditions. The generated films were cut into 1 cm diameter discs using a cork borer.

### 3.2. Characterization of Captopril ODFs

#### 3.2.1. Dimension, Drug Content, and Surface pH Measurements

Thickness and weight were measured for each formulation using six different ODFs from different batches. With the use of a Mitutoyo micrometer from Kanagawa, Japan, the thickness was measured. An analytical balance was used to measure the weight of each film (Mettler Toledo, Zurich, Switzerland).

Six captopril ODFs were dissolved in 100 mL of distilled water, sonicated for 10 min, filtered, appropriately diluted, and determined spectrophotometrically at 227 nm by a Shimadzu 16001 spectrophotometer (Kyoto, Japan). The surface pH of the prepared captopril ODF was determined, as also reported elsewhere [24]. A sample of 500 µL of water was added to captopril ODFs until hydration of the films. The pH was measured using a pH indicator strip (VWR International Ltd., Poole, UK).

##### Folding Endurance

Six captopril ODFs were frequently folded from the center until the films were torn. The number of times the ODF was bent until tearing was the value of folding endurance.

#### 3.2.2. In Vitro Disintegration Time

Captopril ODFs were placed individually into 6 glass tubes of the basket assembly that were immersed in a thermo-stated water bath containing 1 L (L) of phosphate buffer pH 7.4 containing 1% Tween 80 at 37 ± 1 °C using Erweka ZT 121, GmbH, Germany. The basket assembly was raised and lowered at a frequency of 30 cycles/min. The time required to disintegrate/break up was taken as disintegration time.

### 3.3. FTIR and DSC

FTIR investigations were conducted on captopril powder and selected ODFs using an FTIR spectrophotometer (Shimadzu IR-345, Kyoto, Japan). Using a DSC Mettler Toledo Star System, Zurich, Switzerland, DSC investigations were utilized to examine the thermal events of the samples over a temperature range of 25 to 200 °C at a rate of 10 °C/min.

### 3.4. In Vitro Release Study

In vitro captopril release from captopril ODFs and commercially available tablets (Capoten^®^) were studied using the USP dissolution apparatus 1 (basket) type. Phosphate buffer (500 mL) with a pH of 7 and 0.1% Tween 80 made up the medium. The mixture was swirled at a speed of 50 rpm and heated to 37 ± 1 °C. At predetermined intervals of 5, 10, 15, 30, and 60 min, a sample (5 mL) was taken. At 227 nm, the drug content was measured spectrophotometrically, as previously described.

Relative release rates (RRR) at 5, 10, and 15 min were estimated by dividing the cumulative amount at a specific time point by the cumulative amount released from the commercial tablet at the same time. Fitting cumulative release data into three kinetics models allowed for the study of release kinetics (zero-order, first-order, and Higuchi diffusion models).

Zero-order equation:

$$C_{t}=C_{0}+K_{0}t$$

First-order equation:

$$\frac{dct}{dt}=K_{1}C_{0}$$

Higuchi diffusion model:

$$C=K_{H}t^{0.5}$$

where *K*_0_, *K*_1_, and *K_H_* denote the release rate constant for zero-order, first-order, and Higuchi models, respectively. *C*_0_ and *C_t_* are the amounts released at zero and *t* time, respectively.

### 3.5. Scanning Electron Microscopy (SEM)

Drug-free and captopril ODFs were studied using the scanning microscope JEOL-JSM6510LA, JEOL, Tokyo, Japan. Using a gold sputter (SPI-module sputter coater, SPI Supply Inc., West Chester, PA, USA), samples were coated on carbon stubs before being scanned under the microscope at a 5 kV acceleration voltage.

### 3.6. Pharmacokinetics Study

According to the ethics clearance (No. HV19/2020) approved by the Faculty of Pharmacy, Minia University, on 1 September 2020, six male volunteers between the ages of 32 and 45 were recruited for this study. Two groups were formed (three volunteers in each group). Group 1 received a single dose of Capoten^®^ (12.5 mg) tablets, while Group 2 received F2 (12.5 mg). The following time intervals were used to collect blood samples: 0.5, 1, 2, 3, 6, 9, and 12 h. The blood samples were centrifuged at 2000× *g* for 5 min to separate the plasma; the samples were kept at −20 °C.

### 3.7. HPLC Analytical Procedure: Standard Solutions, Calibration Samples, and Quality Control Samples

Stock solutions of captopril and the IS (1000 µg/mL) were prepared in acetonitrile (ACN). Working solutions of captopril (5–500 ng/mL) and the IS (100 ng/mL) were prepared by mixing and diluting with ACN. All FMOC-Cl solutions were prepared in ACN on the same day of analysis. Borate and phosphate buffers were prepared in water and adjusted to the appropriate pH values with sodium hydroxide or phosphoric acid solutions, respectively. All solutions were stored at 4 °C and remained stable for at least 4 weeks. Plasma calibration standards were prepared by spiking 500 µL of blank plasma with working standard solutions. Plasma quality control samples were prepared at three levels: low-QC (15 ng/mL), middle-QC (100 ng/mL), and high-QC (500 ng/mL). Before being injected into the HPLC system, both standard and sample solutions were filtered using a 0.20 mm syringe filter.

#### 3.7.1. Precolumn Derivatization

Volumes of 500 µL of samples or calibration standard solutions and 10 µL of IS were placed in centrifuge tubes, followed by the addition of 50 µL of FMOC-Cl solution (1 mg/mL) and 50 µL borate buffer pH 8.4 (0.1 M), and mixed well. The solutions were kept at 49.5 °C for 23 min to ensure complete derivatization. The excess reagent was removed from the solution by adding 1000 µL n-hexane solution, vortex shaking, and discarding the upper hexane layer. A volume of 10 µL of the reaction mixture was injected into the HPLC system.

#### 3.7.2. Samples Preparation

A sample of 500 µL of plasma (blank or spiked) and another 10 µL IS were placed into 2 mL Eppendorf tubes, followed by 500 µL of the ACN solution for protein precipitation. To promote phase separation, the mixture was vortexed for 1 min and centrifuged at 2000× *g* for 5 min. The upper analyte-enriched acetonitrile phase was retrieved using a glass syringe, transferred to a centrifuge tube, and derivatized as described in Section 3.7.1.

#### 3.7.3. Equipment and Chromatographic System

The chromatographic analyses were performed using a Thermo Scientific UltiMate 3000 HPLC System with an HPG-3400SD solvent delivery pump, a WPS-3000(T)SL analytical autosampler, and a Dionex UltiMate-3000 RS fluorescence detector. The HPLC system control and data processing were performed by computer integration software (Thermo Scientific™ Dionex™ Chromeleon™ 7.2 Chromatography Data System (CDS)).

Chromatographic separation was performed with a Luna omega PS RP-C18 column (100 × 4.6 mm i.d. with 3 µm particle size; Phenomenex, Torrance, CA, USA) equipped with a security guard cartridge. The column oven was kept at 40 °C, and the injection volume was set at 10 µL. The fluorescence detector was set with 265 nm and 315 nm as the excitation and emission wavelengths, respectively, and the mobile phase flow was maintained at 0.7 mL/min. The mobile phase consisted of a mixture of ACN: 30 mM phosphate buffer (pH 3.5) at a 60:40 (*v*/*v*) ratio. It was filtered through a 0.2 mm membrane filter (Phenomenex, USA) using a vacuum filtration unit (Phenomenex, USA) and degassed in an ultrasonic cleaner (Cole-Parmer, Chicago, IL, USA).

### 3.8. Statistical Analysis

GraphPad Prism software version 8.4.3 (San Diego, CA, USA, www.graphpad.com, accessed on 2 May 2023) was used to conduct an unpaired *t*-test and a one-way ANOVA followed by Dunnett’s multiple comparisons test.

## 4. Conclusions

Pediatric hypertension is a serious health issue affecting up to 2% of children. Many antihypertensive drugs have been approved to treat hypertension in children; however, there is a scarcity of dosage forms that can provide accurate dosing for this special patient population. Most of the treatments available for pediatric hypertension are prepared extemporaneously. Oral dispersible films (ODF) of captopril were successfully prepared from a combination of alginate, gelatin, PVA, and PVP with high palatability and fast disintegration (approx. 1 min for the optimized F2). Pharmacokinetics parameters were measured and recorded for the optimized ODF and indicated less plasma peaking (64 ng/mL vs. 101 ng/mL), prolonged plasma levels (T_max_ = 3.59 h vs. 0.66 h), and consistent bioavailability (AUC = 1000 vs. 701 ng min/mL) compared with the commercial immediate-release tablets, respectively. This warrants the use of the proposed formulations for the treatment of pediatric hypertension.

## Figures and Tables

**Table 1:** Measured parameters for captopril oral films with different compositions: drug content, surface pH, folding durability, thickness, weight, and disintegration time. Data are presented as mean ± SD, n = 6.

Formulation Symbol	F1	F2	F3	F4
Thickness (µm)	400.0 ± 8.0	500.0 ± 5.0	450.0 ± 5.5	380.0 ± 5.0
Weight (mg)	38.5 ± 2.0	50.0 ± 1.0	50.0 ± 2.0	38.5 ± 0.5
Drug content (mg)	11.5 ± 1.1	12.5 ± 0.4	12.35 ± 0.5	11.0 ± 1.0
Surface pH	7.5	7.0	7.0	7.5
Folding endurance	7.0 ± 1.0	5.0 ± 2.0	10.0 ± 2.0	2.0 ± 1.0
Disintegration time (min)	2.0 ± 1.0	1.0 ± 0.5	8.0 ± 2.0	10.0 ± 2.0

**Table 2:** Release parameters (relative release rate (RRR) at 5, 10, and 15 min) and release kinetics estimated for the prepared captopril ODFs. Data are presented as mean ± SD, n = 3.

Release Parameters	RRR_5min_	RRR_10min_	RRR_15min_	Zero Order	First Order	Higuchi Model
Formulation symbol				R^2^	R^2^	R^2^
F1	3.25 ± 0.30	4.13 ± 0.17	1.35 ± 0.07	0.94	0.91	0.99
F2	4.13 ± 0.17	4.21 ± 0.30	1.35 ± 0.08	0.83	0.79	0.97
F3	2.35 ± 0.20	2.70 ± 0.14	1.35 ± 0.03	0.91	0.84	0.99
F4	3.25 ± 0.20	3.90 ±0.14	1.35 ± 0.03	0.82	0.79	0.94
Capoten^®^	-	-	-	0.84	0.77	0.98

**Table 3:** Analytical performance of the developed HPLC method.

Linearity Range (ng/mL) ^a^	Determination Coefficient (r^2^)	Equation	LOD (ng/mL)	LOQ (ng/mL)
5–500	0.9998	y = 0.0108x − 0.0075	4.0	13.4

^a^ Peak area ratio of the analyte/IS versus the corresponding concentration (ng/mL).

**Table 4:** Intra- and inter-day method accuracy and precision at three spiked levels.

Concentration (ng/mL) ^a^	Intra-Day Assay (n = 6)		Inter-Day Assay (n = 6)	
	% Recovery	Precision (% RSD) ^b^	% Recovery	Precision (% RSD) ^b^
15 (LQC)	98.48	1.23	99.08	1.07
100 (MQC)	102.01	1.88	100.88	1.45
500 (HQC)	99.29	1.49	101.21	2.12

^a^ Concentrations: LQC, low quality control; MQC, middle quality control; and HQC, high quality control; ^b^ RSD, relative standard deviation.

**Table 5:** The pharmacokinetic parameters of captopril in plasma. Data are presented as mean ± SD, n = 3.

Pharmacokinetic Parameter	Value (Mean ± SD)	
	Captopril^®^ Tablet	Captopril Film (F2)
C_max_ (ng/mL)	101.3 ± 3.2	63.66 ± 5.65
T_max_ (h)	0.66 ± 0.07	3.59 ± 0.80
K_a_ (h^−1^)	3.3 ± 0.75	0.66 ± 0.45
K_el_ (h^−1^)	0.51 ± 0.04	0.11 ± 0.07
t_1/2_ (h)	1.36 ± 0.13	7.8 ± 2.65
AUC (ng min/mL)	701 ± 39	1000 ± 250

**Table 6:** Composition of the prepared modified-release oral films.

Ingredients	F1	F2	F3	F4
Captopril (mg)	150	150	150	150
Sucralose (mg)	150	150	150	150
PVA 5% (g)	5	5	10	5
Gelatin 5% (g)	10	15	10	25
SA 5% (g)	15	10	10	-
Glycerin (mg)	544.5	544.5	544.5	544.5

## Data Availability

Upon request.
